# Roles of Gastric Emptying and Gastrointestinal Transit Following Ileal Interposition in Alleviating Diabetes in Goto-Kakizaki Rats

**DOI:** 10.3389/fendo.2022.849923

**Published:** 2022-03-02

**Authors:** Yanmin Wang, Xiaomei Guo, Dong Sun, Ghassan S. Kassab

**Affiliations:** ^1^ California Medical Innovations Institute, San Diego, CA, United States; ^2^ Department of General Surgery, Qilu Hospital of Shandong University, Jinan, Shandong, China

**Keywords:** ileal interposition, type 2 diabetes, glucagon-like peptide-1, Goto-Kakizaki rat, gastric emptying, gastrointestinal transit

## Abstract

**Objective:**

This study aimed to determine the change of gastrointestinal (GI) emptying time after ileal interposition (IT) and elucidate the role of altered GI peristalsis in diabetic control.

**Materials and Methods:**

Twelve male Goto-Kakizaki rats were randomly divided into IT and sham groups. Body weight and food intake were recorded. Oral glucose tolerance test (OGTT), insulin tolerance test (ITT), plasma glucagon-like peptide-1 (GLP-1), and gastric emptying were measured at baseline and 4 and 8 weeks after operation. At 9 weeks postoperatively, the rats in the IT group were given atropine which can suppress the emptying of stomach and upper intestine, while sham rats were given metoclopramide (to expedite gastric emptying) for 1 week. At week 10 postoperatively, OGTT and GLP-1 were detected. The intestinal transit was tested at postoperative 12 weeks.

**Results:**

No differences were found between groups at baseline. After operation, the IT rats had lower body weight than sham rats. At 4 and 8 weeks postoperatively, the IT group showed better OGTT and ITT, with significantly elevated GLP-1 relative to sham. After administration of the GI motility drugs, however, the effect of diabetic control for the two groups became similar. The GI transit after IT was significantly slower than sham at all tested time points.

**Conclusions:**

Although IT inhibits the GI transit time, the earlier interaction between undigested nutrients and interpositioned ileum promotes gut hormone secretion and thus reduces body weight and alleviates hyperglycemia. A decrease of GI transit of IT rats exacerbates the antidiabetic effects.

## Introduction

Diabetes is a worldwide health crisis that affects morbidity and mortality (1) where 90% of diabetics suffer from type 2 diabetes mellitus (T2DM) (2). Worse yet, the prevalence of T2DM has been dramatically growing (1, 3, 4). Diabetes is a major risk factor of hypertension, cardiovascular disease, renal disorders, stroke, and other diseases.

In recent years, bariatric surgeries have emerged as recommended treatment methods for obesity and T2DM. Bariatric procedures are considered more effective for T2DM resolution than conventional medical therapy, especially for refractory diabetics (5, 6); however, the precise mechanisms are not fully understood. Among the various hypotheses, gastrointestinal (GI) transit seems to play a role in the antidiabetic effect, but the conclusion is not solid. It has been hypothesized that hyperglycemic control after Roux-en-Y bypass (RYGB) is mediated by the time needed to empty nutrients from the stomach to the distal intestine (7, 8). Vertical sleeve gastrectomy (VSG) is being reconsidered not only a restrictive procedure but also as a promoter of GI motility that enhances glucagon-like peptide-1 (GLP-1) release (7. 9), in agreement with “hindgut hypothesis” (10). On the other side, studies in adjusted gastric banding (AGB) reported that AGB has no influence or even delays gastric emptying (11, 12). Some GI devices were also reported to decrease GI emptying although the bariatric effects are good (13).

In this controversial issue, studies involving GI emptying time changes after other bariatric or metabolic procedures such as ileal interposition (IT) are lacking. IT is also widely used in diabetic research, and the effect of IT surgery also supports the hindgut hypothesis (10, 14), as the anatomic structure after IT causes an earlier contact between luminal nutrients and the interposed distal gut. It is not clear whether the increased gut peptides after IT, however, are also attributed to faster chyme delivery. Decades ago, a study by Ohtani et al. (15) showed that IT (referred to as “ileojejunal transposition”) inhibits postprandial gastric emptying because of “ileal break”, without affecting intestinal transit in dogs. There have not been any other reports on the change of GI transit after IT. Moreover, the role of GI transit in diabetic control following IT remains unclear.

Here, we tested glucose indexes, insulin tolerance, and GLP-1 concentration, under different GI peristalsis conditions, to study the relationship between glucose homeostasis and changed GI transit, seeking to understand the role of GI transit in alleviating diabetes after IT, in Goto-Kakizaki (GK) rat model (16), the most widely used nonobese animal model of T2DM. The implications and limitations of the studies are enumerated.

## Materials and Methods

### Animals

Twelve-week-old male GK rats were purchased from Charles River Laboratories (Massachusetts, USA). All animals were housed in individual cages under constant ambient temperature and humidity in a 12-h light/dark cycle room of the California Medical Innovations Institute (San Diego, CA, USA). Animals were given free access to tap water and fed with a commercial diet (18% protein; Envigo, Livermore, CA, USA) during the period of the study, unless otherwise specified. The animal experiments were approved by the Institutional Animal Care and Use Committee of California Medical Innovations Institute.

### Surgical Procedures

After an acclimation of 2 weeks, twelve GK rats were randomly assigned into the IT group and sham group (*n* = 6 for each). Before operations, rats were fed 10% Ensure solution (Abbott Laboratories, Des Plaines, IL, USA) for 2 days and fasted for 12 h. Under anesthesia with isoflurane (2% with 1 L/min oxygen), the rats were given IT or sham operations, respectively.

**Figure 1:**
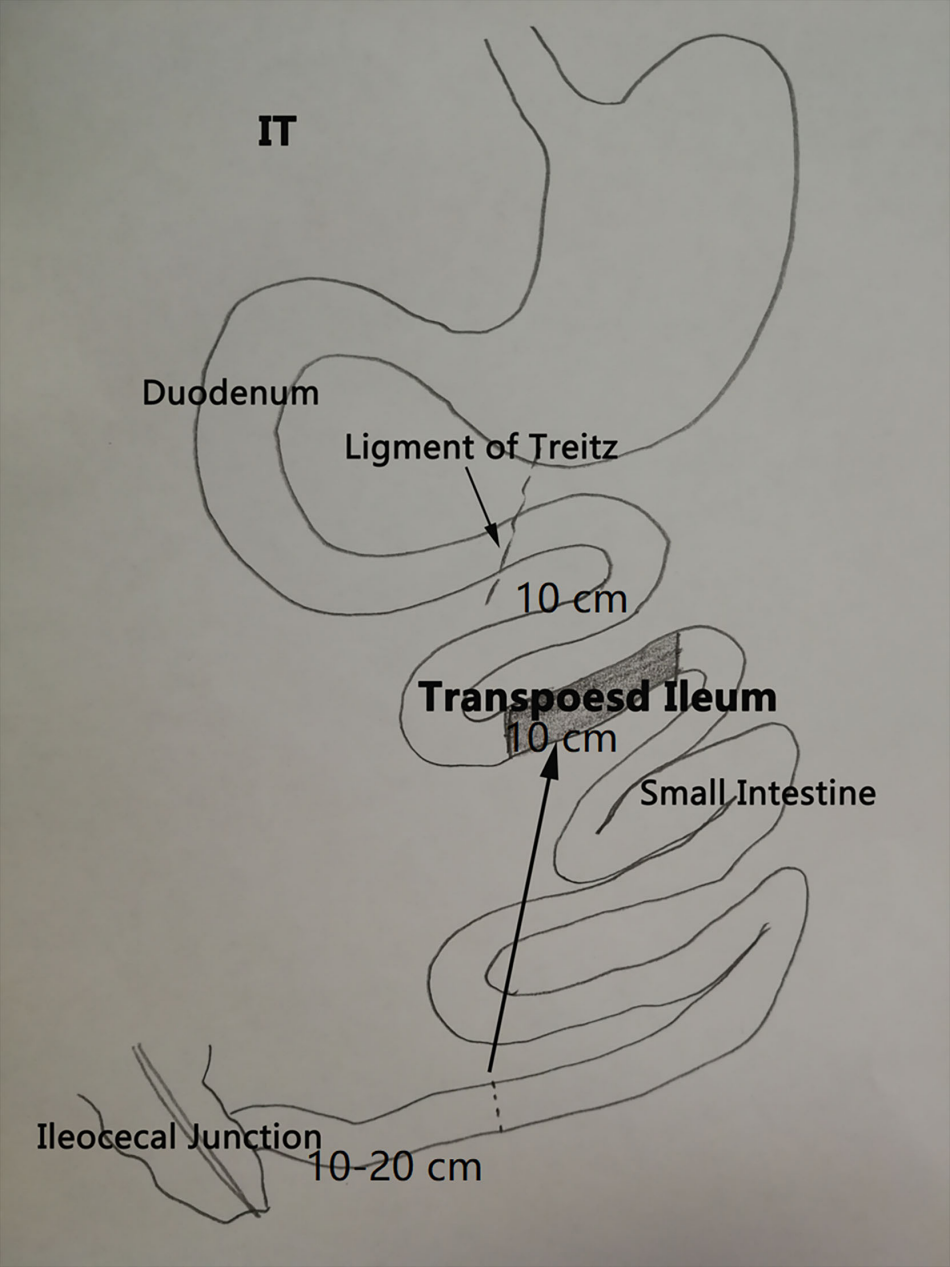
Schematic diagram of ileal interposition.

As previously described (17), IT (**Figure 1**) involves removal of 10 cm of ileum at 10–20 cm proximal to the ileocecal junction and a transection of the jejunum 10 cm distal to the ligament of Treitz. The resected ileal segment was then interposed at the transection site in the original peristaltic direction, and three end-to-end anastomoses were performed using 6-0 Prolene suture. Sham procedure included the same transections of small intestine, but reanastomoses were made *in situ*. The operative time of sham was prolonged similar to that of the IT group, in order to normalize the anesthetic and surgical stress in both groups.

During the first 24 h following operation, the rats were only given water, with 2 ml 5% glucose solution and 2 ml normal saline in subcutaneous injection for rehydration. The rats were then fed 10% Ensure for 3 days, followed by regular diet. Body weight and calorie intake were recorded three times per week for 10 weeks following surgery.

### Experimental Protocol

The experimental protocol and timelines are shown as listed in **Table 1**. At baseline as well as at 4 and 8 weeks postoperatively, oral glucose tolerance test (OGTT), insulin tolerance test (ITT), total glucagon-like peptide-1 (GLP-1), and gastric emptying were measured. The detailed specifications of these indexes will be described further below.

**Table 1:** Timeline of the experiments.

Week	IT group (*N* = 6)	Sham-IT group (*N* = 6)
−2	Acclimation	
0	Baseline	
	IT surgery	Sham surgery
1	Postoperative recovery	
2	Body weight, food intake	
4	Body weight, food intake, OGTT, ITT, GLP-1, acetaminophen	
8	Body weight, food intake, OGTT, ITT, GLP-1, acetaminophen	
9	Atropine, once daily	Metoclopramide, once daily
10	Stop atropine	Stop metoclopramide
	Body weight, food intake, OGTT, GLP-1	
12	Measurement of GI transit	

If not specified otherwise, sham group followed the same protocol as IT group.

To further study the role of GI emptying time, GI motility medications were given. At week 9 postoperation, the IT rats were intraperitoneally injected with atropine (1 mg/kg; Sigma-Aldrich, St. Louis, MO, USA) for 7 days, which can be used to inhibit the movement of stomach and upper intestine. During the same period, sham rats were given an intraperitoneal injection of metoclopramide (1 mg/kg; Sigma-Aldrich, St. Louis, MO, USA), which is known to accelerate gastric emptying. At postoperative week 10 (atropine and metoclopramide had already been used daily for 1 week), OGTT and GLP-1 were also tested.

At postsurgery week 12 (scheduled termination; the administration of medicines had been stopped for 2 weeks), the nutrient transit in GI tract was measured using validated methods (7, 17). Toluidine blue O (Sigma-Aldrich, St. Louis, MO, USA) was mixed with Ensure solution and then orally administered to the fasted rats (0.5 ml/100 g). Thirty minutes later, a laparotomy was performed under isoflurane inhalation. The overall length of small intestine and the distance between the distal end of the dye and the ileocecal junction were measured.

### Glucose Evaluation

The test of blood glucose was always undertaken in duplicate from tail vein by a portable glucometer (Roche Diagnostics, Mannheim, Germany). In OGTT, after an overnight fast, the rats were orally administrated 20% glucose (1 g/kg), and the blood sugar was measured at 0, 15, 30, 60, and 120 min. Meanwhile, blood samples were collected. The serum total GLP-1 was measured using an ELISA kit (Millipore, Burlington, MA, USA). In ITT (18), blood glucose was measured in conscious and fasted rats, and then 10, 30, 60, 90, 120, and 180 min after intraperitoneal injection of insulin solution (0.5 IU/kg; Sigma-Aldrich, St. Louis, MO, USA).

### Gastric Emptying Measurements

The measurement of gastric emptying was based on standard methods (7). Briefly, acetaminophen (100 mg/kg) was added to 3 ml Ensure solution and then perorally infused to overnight-fasted rats. Blood samples were collected at 15, 30, 45, and 60 min after intragastric gavage. The concentration of serum acetaminophen, which is not absorbed in the stomach (thus used as indicator of gastric emptying), was detected by spectrophotometric method under an absorption spectrum at 590 nm (19).

### Statistical Analysis

The results were expressed as mean ± standard deviation (SD). Areas under the curves (AUC) for OGTT (AUC_OGTT_) and ITT (AUC_ITT_) were calculated by trapezoidal integration. Differences between groups over time were evaluated by two-way analysis of variance (ANOVA) or repeated measures ANOVA; differences between the two groups at a specific time point were calculated using Student’s *t*-test. Homogeneity of variance was confirmed by levene statistic test, and Friedman test was performed for heterogeneity. All statistical analyses were performed by SPSS19.0 software. *p* < 0.05 was considered statistically significant.

## Results

Before operation, all rats had the same levels of body weight, food intake, serum total GLP-1, and acetaminophen. All procedures were successfully performed, and all rats survived till the scheduled time of termination.

### Body Weight and Food Intake

**Figure 2:**
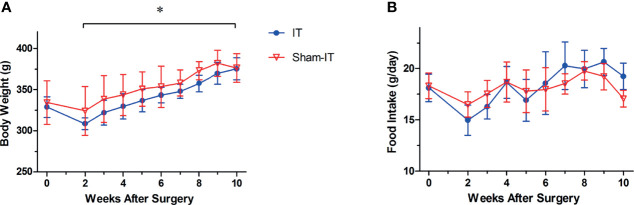
Body weight and food intake after operation. *N* = 6 in each group. ^*^ *p* < 0.05. **(A)** Body weight. IT group exhibited less body weight than sham group after surgery (two-way ANOVA: *p* < 0.001). **(B)** Food intake. No difference was found in food intake between the two groups (two-way ANOVA: *p* = 0.454).

Postoperatively, rats in the sham group showed greater body weight than IT rats (**Figure 2A**; **Supplementary Material**). After injection of atropine/metoclopramide, however, the body weight of IT and sham rats became similar (*p* > 0.05). No difference was observed in food intake between the two groups, as shown in **Figure 2B**.

### Glucose Homeostasis and Insulin Sensitivity

**Figure 3:**
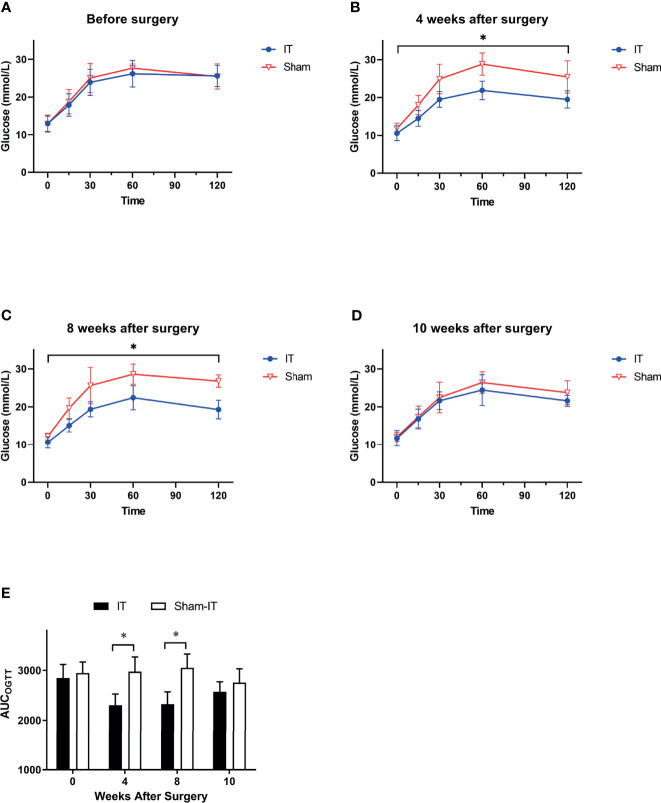
OGTT results at different time points. *N* = 6 in each group. ^*^ *p* < 0.05. **(A)** OGTT before surgery. No difference was shown between IT and sham groups (two-way ANOVA: *p* = 0.569). **(B)** OGTT at 4 weeks after surgery. Glucose level following oral gavage was significantly lower in IT group (two-way ANOVA: *p* = 0.002). **(C)** OGTT at 8 weeks after surgery. IT rats had significantly lower glucose level than sham rats (two-way ANOVA: *p* = 0.001). **(D)** OGTT at 10 weeks after surgery. IT rats and sham rats showed similar OGTT results (two-way ANOVA: *p* = 0.269) after administration of GI motility medications. **(E)** AUC_OGTT_. Before surgery, there was no difference in AUC_OGTT_ between groups (*t*-test, *p* = 0.497). At 4 and 8 weeks after surgery, AUC_OGTT_ was significantly decreased in IT group (*t*-test, *p* = 0.001 of both). At postoperative 10 weeks, however, the difference became not significant (*t-*test, *p* = 0.215).

**Figures 3A–D** shows OGTT data at weeks 0, 4, 8, and 10. There was no significant difference between groups before surgery. At 4 and 8 weeks postoperatively, OGTT was significantly improved in IT rats as compared with sham. These findings were also supported by the data of AUC_OGTT_ (**Figure 3E**). After the use of GI motility pharmacologics (at 10 weeks after surgery), however, the gaps in OGTT curves and AUC_OGTT_ between IT and sham became smaller, with no statistical differences between the two groups.

**Figure 4:**
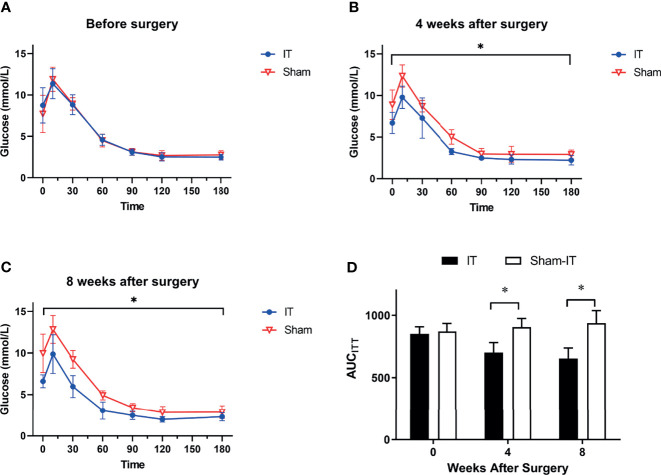
ITT results at different time points. *N* = 6 in each group. ^*^ *p* < 0.05. **(A)** ITT before surgery. No difference was found between groups (two-way ANOVA: *p* = 0.993). **(B)** ITT at 4 weeks after surgery. IT rats showed significantly improved ITT as compared with sham rats (two-way ANOVA: *p* = 0.001). **(C)** ITT at 8 weeks after surgery. ITT data of IT rats was significantly better than sham (two-way ANOVA: *p* < 0.001). **(D)** AUC_ITT_. No difference was observed in AUC_ITT_ between IT and sham groups before operation (*t*-test, *p* = 0.605). At postoperative 4 weeks, AUC_ITT_ were significantly decreased in IT group (*t*-test, *p* = 0.001). At 8 weeks postoperatively, the difference became more significant (*t*-test, *p* < 0.001).

As shown in **Figure 4A**, there were no differences in ITT between the IT and sham groups prior to operation. At postoperative 4 and 8 weeks, glucose levels in response to insulin injection were significantly decreased in the IT group as compared with sham, suggesting improvements of glucose tolerance and insulin tolerance (**Figures 4B, C**). The results were consistent with AUC_ITT_ calculation as shown in **Figure 4D**.

### Plasma Total GLP-1

**Figure 5:**
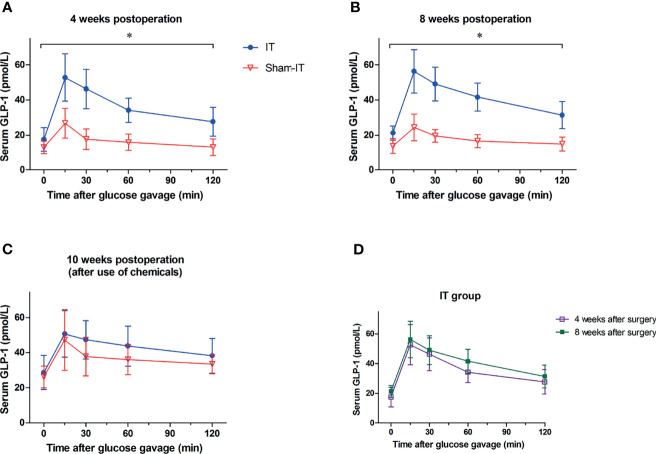
The levels of serum total GLP-1 after surgery. *N* = 6 in each group. ^*^ *p* < 0.05. **(A)** Glucose-stimulated GLP-1 levels at postoperative 4 weeks. As compared with sham, GLP-1 level was significantly higher in IT group at 4 weeks postoperatively (repeated measures ANOVA: *p =* 0.001 IT vs. sham, *p* < 0.001 between time points, *p* < 0.001 of interaction). **(B)** Glucose-stimulated GLP-1 levels at 8 weeks postoperatively. GLP-1 level was significantly higher in IT group at postoperative 8 weeks in comparison with sham group (repeated measures ANOVA: *p* < 0.001 IT vs. sham, *p* < 0.001 between time points, *p* < 0.001 of interaction). **(C)** Glucose-stimulated GLP-1 levels at postoperative week 10. At 10 weeks after operation, because of the use of chemicals, GLP-1 levels were comparable between IT and sham groups (repeated measures ANOVA: *p =* 0.309 IT vs. sham, *p* < 0.001 between time points, *p =* 0.680 of interaction). **(D)** Glucose-stimulated GLP-1 levels of IT group. GLP-1 concentration at 8 weeks postoperatively was not significantly higher than that at 4 weeks postoperatively (repeated measures ANOVA: *p =* 0.358 IT vs. sham, *p* < 0.001 between time points, *p =* 0.885 of interaction).

At postoperative weeks 4 and 8, relative to sham, the IT group exhibited significantly higher plasma concentration of GLP-1 (**Figures 5A, B**). At 10 weeks after surgery, following administration of GI dynamic drugs, however, the IT and sham groups showed no difference in GLP-1 concentration (**Figure 5C**). For the IT group, GLP-1 level seemed elevated in week 8 as compared with week 4 postoperatively, but there is no statistically significant difference (**Figure 5D**).

### Gastric Emptying and Intestinal Transit

**Figure 6:**
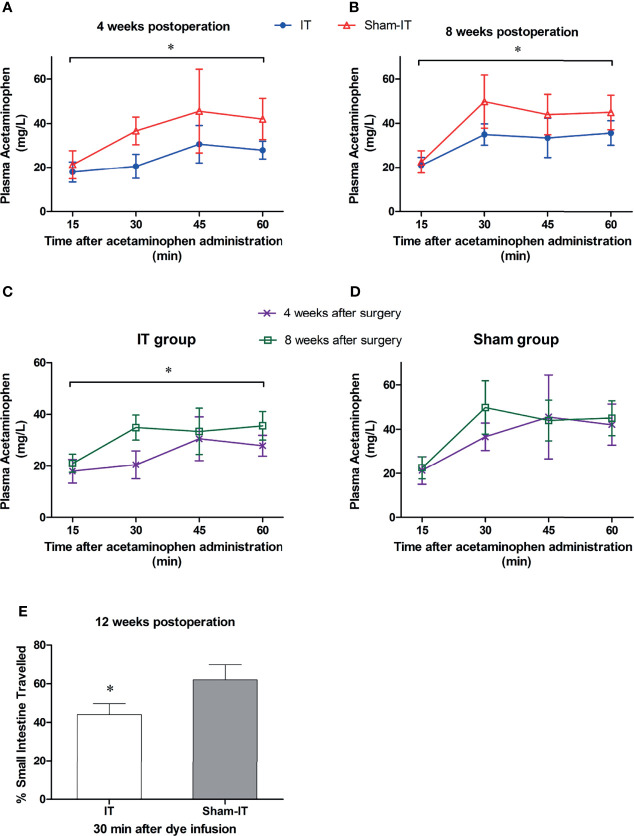
The tests of GI motility after surgery. *N* = 6 in each group. ^*^ *P* < 0.05. **(A)** Plasma levels of acetaminophen at postoperative 4 weeks. The concentrations of acetaminophen were significantly lower in IT group than in sham group (Friedman test: *p* < 0.001). **(B)** Acetaminophen levels at postoperative 8 weeks. The concentration was significantly higher in sham than in IT group (repeated measures ANOVA: *p =* 0.010 IT vs. sham, *p* < 0.001 between time points, *p =* 0.121 of interaction). **(C)** Plasma levels of acetaminophen in IT group. The concentration of acetaminophen was higher at 8 weeks postoperatively than at 4 weeks (repeated measures ANOVA: *p =* 0.021 IT vs. sham, *p* < 0.001 between time points, *p =* 0.015 of interaction). **(D)** Plasma levels of acetaminophen in sham group. The levels did not differ over time (repeated measures ANOVA: *p =* 0.249 IT vs. sham, *p* < 0.001 between time points, *p =* 0.302 of interaction). **(E)** Intestinal transit (expressed as percentage of intestine travelled) at 30 min after toluidine blue O infusion. The dye travelled shorter in intestinal tract in IT compared to sham group (*t*-test, *p* = 0.001).

As shown in **Figure 6A, B**, the serum levels of acetaminophen following IT were significantly lower than that of the sham group at 4 and 8 weeks postoperatively, indicating a slower gastric emptying. For the IT group, the speed of gastric emptying was markedly increased in postoperative week 8, as the concentration of acetaminophen was higher than that of week 4 (**Figure 6C**). In the sham group, the emptying of stomach was not changed over time (**Figure 6D**). **Figure 6E** shows that 30 min after toluidine blue O gavage, the dye travelled significantly shorter in the intestinal tract in IT in comparison with sham (at 12 weeks after surgery).

## Discussion

Studies of IT have been performed in rats for over 30 years (20). In recent years, IT has also been used in patients, with satisfactory outcomes (21–23). Unlike RYGB and VSG, IT does not involve gastrectomy; i.e., it is a “pure” intestinal operation which is suitable for GI research. The hindgut hormones are considered vital mediators of diabetic control following IT (17). Although it is known that the rapid delivery of mixed chyme to transposed ileum promotes the secretion of hindgut hormones, the role of GI emptying in accelerating these hormones and alleviating hyperglycemia post-IT requires further investigations.

To minimize the impact of different body weight on blood glucose and focus on the weight loss independent mechanism (24), we utilized nonobese animal model, i.e., GK rats, for the study. GK diabetic rats are characterized by insulin resistance and insufficient pancreatic beta cell function, which can appropriately simulate human T2DM (18, 25, 26). Using GK rat model, our experiments illustrate the GI transit time to be suppressed after IT, and gastric emptying gradually recovered over time. This represents the first study of the relationship between GI emptying and diabetic control after IT.

Although it is believed that sustainable weight loss benefits diabetic alleviation (27), previous data on weight change after IT are not unified. Some literature (14, 28) shows that IT does not affect body weight, while some others (29, 30) found a decrease in weight gain in IT rats. The paradox may be explained by the differences of studied strains, housing environments, operative details, etc. In our study, IT induced reduction of body weight, albeit the amount of food intake was not different. A study by Gaitonde et al. (14) also revealed an unchanged level of food intake after IT. Given that IT does not reduce the absorption area of gut, one reasonable speculation is that IT surgery may augment energy expenditure (31), in agreement with some literature on RYGB (32, 33), resulting in disparity of body weight. Another plausible explanation is that metabolic surgery reprograms the whole-body metabolism to maintain a reduced body weight, rather than lowering caloric intake (34).

Based on our data, glucose tolerance and insulin resistance were both ameliorated after IT, suggesting improved glucose homeostasis. Similarly, Yan et al. (30) and Culnan et al. (35) came to the same conclusion in GK rats and Zucker fatty rats, respectively. We speculate the increased GLP-1 contributes to the remission of glycometabolism (consistent with the hindgut theory), as GLP-1 has effects in improving beta cell function, insulin secretion, and insulin sensitivity (36, 37). In addition, GLP-1 can slow down gastric emptying and intestinal transit (37). Confirmatory to these observations, the movements of both stomach and small intestine were decreased in our IT group. Obviously, the early exposure of chyme is due to the short pathway rather than a rapid transit.

The role of GI emptying and transit was studied at 10 weeks postoperatively. After using atropine in IT rats and metoclopramide in sham rats, it can be speculated that the GI transit of IT decreased while that of sham increased since GI emptying can be inhibited by atropine (7) and promoted by metoclopramide (38). As a result, GLP-1 secretion of the two groups became equalized after the administration of the pharmaceuticals. Consequently, the body weight became comparable, and the differences in glucose tolerance decreased. In other words, increase in GI transit in sham can also be helpful to ameliorate diabetes, while slowing down the GI movement after IT can deteriorate glycemic control. Diabetic rats were found to show gastric hypomotility (39). Furthermore, Bharucha et al. (40) indicated that delayed gastric emptying in diabetes can affect hyperglycemia, which is confirmative of our study. Taken together, the decrease of GI transit following IT may be the result or the negative feedback of increased hindgut hormones, rather than a favorable outcome of diabetic alleviation. The “earlier contact” caused by IT promotes GLP-1 secretion and further changes body weight, glycometabolism, etc., which can be partly blocked by slower GI transit.

One interesting finding is that at 8 weeks postoperatively, the gastric emptying in the IT group was accelerated as compared with postoperative week 4, but it did not lead to further improvement of glucose metabolism or significant increase of GLP-1. This suggests that the acceleration of gastric emptying is not the key factor in diabetic control after IT. Further studies are needed to shed additional light on this issue.

Our study has some limitations. First, we did not test GI motility by scintigraphy, which is more accurate than acetaminophen absorption test and dye measurement, because of the need for complex methodology. Second, the role of other hormones, the measurement of energy expenditure, and the influence of drugs under longer-term application need to be further investigated. Third, gastric emptying and GI transit rate of rats may be different from that of humans, and hence the findings need to be validated in larger animals before translation. Gender-based differences also need to be explored in the future.

In conclusion, IT reduced body weight and alleviated diabetes in GK rats. The increased secretion of GLP-1 may at least partly contribute to the remission of T2DM. However, the inhibition of GI emptying is likely to disable the hypoglycemic effect caused by IT, while the acceleration of GI transit seems advantageous to achieve euglycemia. Following IT, the alteration of GI transit seems secondary to the operation instead of the cause of hyperglycemic control.

## Data Availability Statement

The raw data supporting the conclusions of this article will be made available by the authors, without undue reservation.

## Ethics Statement

The animal study was reviewed and approved by the Institutional Animal Care and Use Committee of California Medical Innovations Institute.

## Author Contributions

YW, XG, and DS performed animal procedures and experiments and data collection. YW and GK designed the study. All authors participated in drafting the manuscript. All authors listed have made a substantial, direct, and intellectual contribution to the work and approved it for publication.

## Funding

This work was supported by 3DT Holdings. The authors declare that 3DT Holdings was not involved in the study design, collection, analysis, interpretation of data, the writing of this article or the decision to submit it for publication.

## Conflict of Interest

GK is founder of 3DT Holdings.

The remaining authors declare that the research was conducted in the absence of any commercial or financial relationships that could be construed as a potential conflict of interest

## Publisher’s Note

All claims expressed in this article are solely those of the authors and do not necessarily represent those of their affiliated organizations, or those of the publisher, the editors and the reviewers. Any product that may be evaluated in this article, or claim that may be made by its manufacturer, is not guaranteed or endorsed by the publisher.
