# Supplementary material for: Roles of Gastric Emptying and Gastrointestinal Transit Following Ileal Interposition in Alleviating Diabetes in Goto-Kakizaki Rats
Source: Front Endocrinol (Lausanne). 2022 Mar 2;13:849923. doi: 10.3389/fendo.2022.849923 (PMC8924662; doi:10.3389/fendo.2022.849923)
Supplement: Supplementary file 1 [file Table_1.docx]

Body weight of the rats (g)

|  | IT group (N=6) | Sham group (N=6) |
| --- | --- | --- |
| Week 0 | 329 ± 12.6 | 334 ± 26.6 |
| Week 2 | 308 ± 7.2 | 324 ± 29.4 |
| Week 3 | 322 ± 15.1 | 339 ± 28.5 |
| Week 4 | 330 ± 15.3 | 344 ± 24.9 |
| Week 5 | 337 ± 13.5 | 351 ± 20.9 |
| Week 6 | 343 ± 9.4 | 354 ± 25.2 |
| Week 7 | 348 ± 8.4 | 358 ± 15.7 |
| Week 8 | 358 ± 10.2 | 373 ± 10.9 |
| Week 9 | 370 ± 13.3 | 382 ± 15.9 |
| Week 10 | 375 ± 13.3 | 376 ± 17.2 |
